# Prothrombotic autoantibodies targeting platelet factor 4/polyanion are associated with pediatric cerebral malaria

**DOI:** 10.1172/JCI176466

**Published:** 2024-04-23

**Authors:** Iset M. Vera, Anne Kessler, Visopo Harawa, Ajisa Ahmadu, Thomas E. Keller, Stephen T.J. Ray, Terrie E. Taylor, Stephen J. Rogerson, Wilson L. Mandala, Morayma Reyes Gil, Karl B. Seydel, Kami Kim

**Affiliations:** 1 Division of Infectious Disease and International Medicine, Department of Internal Medicine, University of South Florida, Tampa, Florida, USA.; 2 Center for Genomics and Systems Biology, Department of Biology, New York University, New York, New York, USA.; 3 Malawi-Liverpool Wellcome Trust Clinical Research Programme, Blantyre, Malawi.; 4 Biomedical Department, University of Malawi College of Medicine, Blantyre, Malawi.; 5 Blantyre Malaria Project, Kamuzu University of Health Sciences, Blantyre, Malawi.; 6 Oxford Vaccine Group, Department of Paediatrics, University of Oxford, Oxford, United Kingdom.; 7 Institute of Infection, Veterinary and Ecological Sciences, University of Liverpool, Liverpool, United Kingdom.; 8 Department of Osteopathic Medical Specialties, Michigan State University, East Lansing, Michigan, USA.; 9 Department of Medicine (RMH), and; 10 Department of Infectious Diseases, Doherty Institute, The University of Melbourne, Melbourne, Australia.; 11 Academy of Medical Sciences, Malawi University of Science and Technology, Thyolo, Malawi.; 12 Department of Pathology, Albert Einstein College of Medicine and Montefiore Medical Center, Bronx, New York, USA.

**Keywords:** Infectious disease, Microbiology, Autoimmune diseases, Malaria, Platelets

## Abstract

**BACKGROUND:**

Features of consumptive coagulopathy and thromboinflammation are prominent in cerebral malaria (CM). We hypothesized that thrombogenic autoantibodies contribute to a procoagulant state in CM.

**METHODS:**

Plasma from children with uncomplicated malaria (UM) (*n* = 124) and CM (*n* = 136) was analyzed by ELISA for a panel of 8 autoantibodies including anti–platelet factor 4/polyanion (anti-PF4/P), anti-phospholipid, anti-phosphatidylserine, anti-myeloperoxidase, anti–proteinase 3, anti-dsDNA, anti–β-2-glycoprotein I, and anti-cardiolipin. Plasma samples from individuals with nonmalarial coma (NMC) (*n* = 49) and healthy controls (HCs) (*n* = 56) were assayed for comparison. Associations with clinical and immune biomarkers were determined using univariate and logistic regression analyses.

**RESULTS:**

Median anti-PF4/P and anti–PS IgG levels were elevated in individuals with malaria infection relative to levels in HCs (*P* < 0.001) and patients with NMC (PF4/P: *P* < 0.001). Anti–PF4/P IgG levels were elevated in children with CM (median = 0.27, IQR: 0.19-0.41) compared with those with UM (median = 0.19, IQR: 0.14–0.22, *P* < 0.0001). Anti–PS IgG levels did not differ between patients with UM and those with CM (*P* = 0.39). When patients with CM were stratified by malaria retinopathy (Ret) status, the levels of anti–PF4/P IgG correlated negatively with the peripheral platelet count in patients with Ret^+^ CM (Spearman’s rho [*R_s_*] = 0.201, *P* = 0.04) and associated positively with mortality (OR = 15.2, 95% CI: 1.02–275, *P* = 0.048). Plasma from patients with CM induced greater platelet activation in an ex vivo assay relative to plasma from patients with UM (*P* = 0.02), and the observed platelet activation was associated with anti–PF4/P IgG levels (*R_s_*= 0.293, *P* = 0.035).

**CONCLUSIONS:**

Thrombosis mediated by elevated anti-PF4/P autoantibodies may be one mechanism contributing to the clinical complications of CM.

## Abstract 2

Prothombotic anti-PF4/polyanion autoantibodies associated with thrombocytopenia are implicated in pathogenesis of cerebral malaria in children.

## Graphical abstract

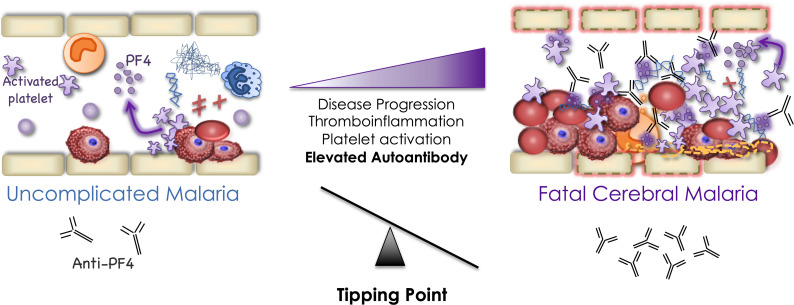

## Introduction

Immunothrombosis is a host defense mechanism where the innate and adaptive immune systems work in conjunction with the hemostatic system (platelets and endothelial cells) to control infection (1). Although it is a protective mechanism, immunothrombosis can quickly advance to pathogenic thromboinflammation resulting in a life threatening pro-coagulant state if unregulated. Hallmark features of thromboinflammation include thrombocytopenia with intravascular coagulation and thrombi formation, uncontrolled activation of innate immune effectors such as neutrophils and monocytes, and endothelial dysfunction (2). Thromboinflammation is a complication observed in noninfectious inflammatory conditions including cardiovascular and autoimmune diseases (3, 4). Thromboinflammatory characteristics are observed in a variety of infections, including those that often lead to sepsis (e.g., *Staphylococcus aureus*, *Escherichia coli*, and *Salmonella* *typhimurium* bacteremia), and viral infections such as SARS-CoV-2, HIV, influenza virus type A, and Dengue virus (DENV) (5, 6), and is also a prominent pathophysiological characteristic in cerebral malaria (CM) (7–13).

CM, caused by infection with *Plasmodium falciparum* parasites, is a neurovascular syndrome associated with decreased consciousness, coma, and seizures and is a leading cause of death in children under the age of five in sub-Saharan Africa (14). Children with CM, as defined by the WHO, present with *P*. *falciparum* infection and are comatose with no other evident etiology of coma. Despite intravenous antimalarial treatment, death still occurs in approximately 15%–20% of patients, suggesting that host processes contribute to poor outcomes. *P*. *falciparum*–infected RBCs (iRBCs) sequester and aggregate in the microcapillaries of lungs, kidneys, liver, intestines, and brain causing obstruction and inflammation (8, 15–17). The initial activation of platelets by iRBCs and subsequent activation and recruitment of monocytes and other immune effectors compromises intravascular integrity (8, 9, 15).

Under inflammatory conditions, damage associated molecular patterns (DAMPs) are produced, becoming potential sources of self-recognition by the immune system (18). Pathogenic autoantibodies that recognize DAMPs can form immune complexes and engage various immune effectors such as complement and Fc receptors, or directly crosslink immune receptors to activate and promote a feedback loop of inflammatory cell activation (19). For example, antibodies that recognize the neutrophil proteins proteinase 3 (PR3) and myeloperoxidase (MPO) are associated with vasculitis and have been shown to stabilize extruded chromatin content from neutrophils called neutrophil extracellular traps (NETs) (20, 21). In addition to entrapping extracellular pathogens, NETs serve as scaffolds for platelet aggregation and clotting that when stabilized by antibodies enhance thrombogenesis (22–24). In cases of heparin-induced thrombocytopenia (HIT), autoantibodies targeting the pleiotropic platelet effector platelet factor 4 (PF4) form immune complexes that bind the platelet Fc γ receptor IIA (FcγRIIA) resulting in platelet activation and further release of autoantigen (PF4) and other procoagulant molecules (25–28). Coronavirus disease 2019 (COVID-19) caused by SARS-CoV2 is also often complicated by hypercoagulation, and autoantibodies specific for phospholipids (PLs), neutrophil proteins, and platelet factors are associated with disease severity and clinical outcome (22, 23, 29–32).

Autoantibodies may contribute to CM pathogenesis via similar mechanisms where systemic and focal inflammatory conditions, including dysregulated platelet activation, NET release, and vascular wall injury offer sources of self-antigens (8, 11, 18, 33–36). Data from both animal models of malaria and humans infected with malaria have suggested that self-reacting antibodies against phosphatidylserine (anti-PS) and against dsDNA (anti-dsDNA) are associated with complications of malaria including anemia and acute kidney injury (37–42).

We hypothesized that procoagulant self-reactive antibodies are elevated in patients with CM and contribute to disease pathogenesis. We measured and compared antibody profiles in Malawian pediatric malaria patients presenting with uncomplicated malaria (UM) or CM and evaluated associations with markers of disease severity and thromboinflammation.

## Results

### Patient characteristics.

**Figure 1:**
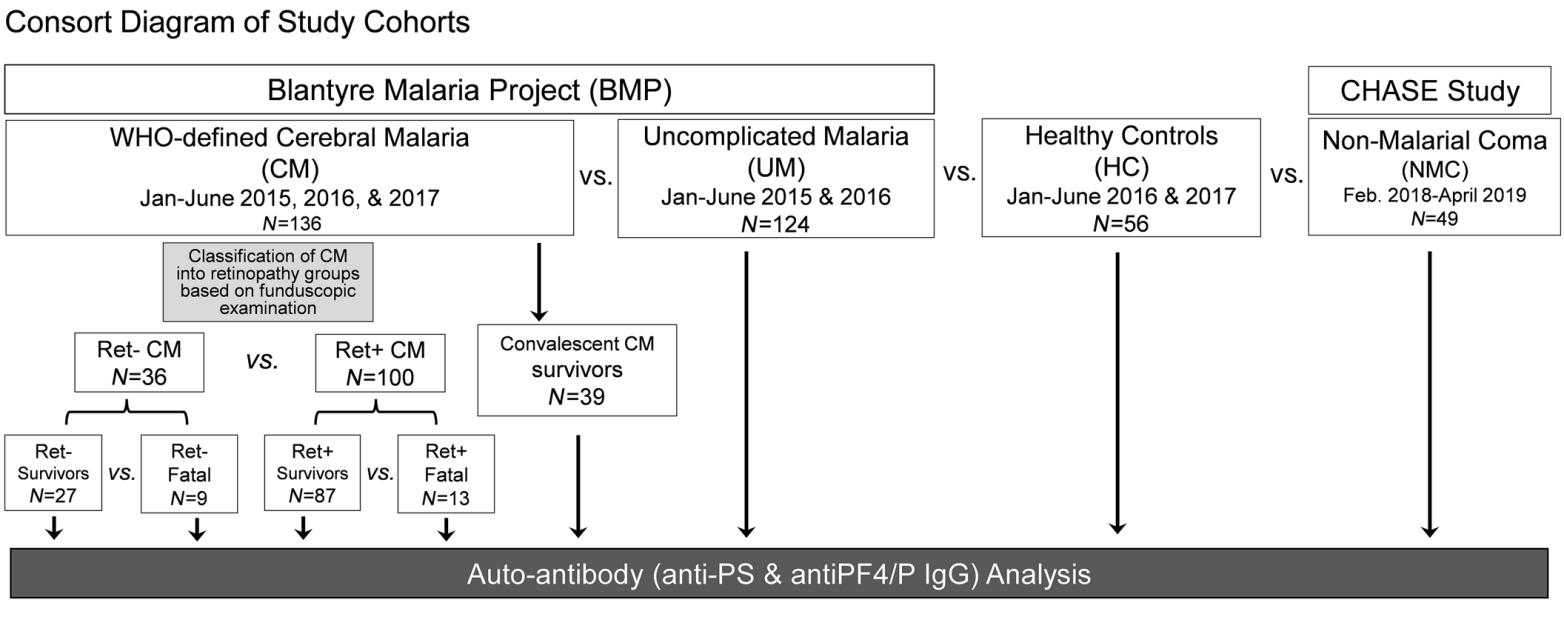
Consort diagram of patient recruitment. Patient recruitment for the BMP included patients with UM or CM. Patients with NMC were recruited under the CHASE study recruitment criteria. Patients with CM were stratified by retinopathy (Ret^–^ or Ret^+^) and by outcome (survivor or fatal). Convalescent CM survivors returned for follow-up assessment 30 days after admission. HCs were recruited from the Ndirande Health Centre while at routine well visits. Plasma samples were collected and analyzed for autoantibody analysis. Feb., February; Jan, January.

Table 1 presents the demographic and clinical characteristics comparing the 4 participant groups: patients with CM (*n* = 136), UM (*n* = 124), patients with nonmalarial coma (NMC) (*n* = 49), and HCs (*n* = 56). The CM group was divided into individuals with (a) retinopathy-positive (Ret^+^ CM, *n* = 100) or retinopathy-negative (Ret^–^ CM, *n* = 36), and those who (b) survived (Ret^+^ CM: *n* = 87; Ret^–^ CM: *n* = 27) or died (Ret^+^ CM: *n* = 13; Ret^–^ CM: *n* = 9) (Figure 1 and Table 1). Abnormal retinal pathology in children presenting with WHO-defined CM improves the specificity of the clinical case definition (43, 44). Ret^–^ CM cases are more heterogeneous, likely representing a mix of milder CM cases and cases of other coma etiology with malaria coinfection.

All groups were analyzed for statistical differences in demographic or clinical characteristics relative to Ret^+^ CM. Children with Ret^–^ CM had lower levels of *P*. *falciparum* histidine-rich protein 2 (*Pf* HRP2) (469 vs. 1,496 ng/mL, *P* = 0.001), but higher platelet counts (148 vs. 60 × 10^3^ cells/μL, *P* < 0.0001) compared with Ret^+^ CM children (Table 1). UM cases presented with increased hemoglobin (HgB) levels (10.0 vs. 7.9 g/dL; *P* = 0.0001), increased platelet count (304 × 10^3^ vs. 60 × 10^3^ cells/μL; *P* < 0.0001), and lower *Pf* HRP2 levels (60 vs. 1,496 ng/mL, *P* < 0.0001) relative to Ret^+^ CM children.

The NMC control patients had a significantly higher HgB (10 vs. 8 g/dL, *P* < 0.0001) and platelet count (330 vs. 60 × 10^3^ cells/μL, *P* < 0.0001) relative to Ret^+^ CM (Table 1). Among the cases of children presenting in coma (NMC and CM), mortality rates were comparable (NMC, 12% vs. Ret^+^ CM, 13%): *P* = 0.897, NMC vs. Ret^–^ CM, 25%: *P* = 0.128). NMC patients had a lower median score on the Blantyre coma scale (BCS), with a higher proportion of patients having a BCS score equal to or less than 1 compared with Ret^+^ CM children (60% vs. 41%, *P* = 0.04; Table 1).

### Analysis of prothrombotic autoantibodies in malaria patients.

In this analysis, levels of a select panel of circulating antibodies in plasma samples were compared between UM and CM patients. Patient plasma was analyzed via ELISA for a panel of IgG autoantibodies (anti-PL, anti-PS, anti-cardiolipin (anti-CL), anti–β-2-glycoprotein I (anti-β2GPI), anti-DNA, anti-PF4/P, anti-MPO, and anti-PR3) associated with thrombogenesis (Supplemental Figure 1; supplemental material available online with this article; https://doi.org/10.1172/JCI176466DS1). We also quantified levels of circulating immune complexes (CICs) in plasma samples as antibody-antigen immune complexes contribute to immunopathology in various autoimmune diseases (45) (Supplemental Figure 2). Of the eight autoantibodies analyzed, only antibodies against the PF4-polyanion antigen (anti-PF4/P) were significantly elevated in CM compared with UM cases (median OD: 0.27 [0.19–0.41] vs. 0.19 [0.14–0.22]; *P* < 0.0001) (Supplemental Figure 1H). Levels of the remaining autoantibodies analyzed were not significantly different between UM and CM (Supplemental Figure 1, A–G, and Table 2). In some patients with malaria, anti-PF4/P IgG levels were elevated above the assay clinical cutoff point (OD = 0.4) for HIT diagnosis, with a higher proportion of levels above the cutoff point observed in patients with CM relative to those with UM (27% of CM vs. 1.6% of UM; Supplemental Figure 1H and Table 2). The levels of CICs above the assay clinical cutoff point (CIC >4 μg/mL) were also observed in some individuals, but neither the prevalence nor the median values differed significantly between the UM and CM groups (74% of CM, 4.84 pg/mL [4.0–7.3] vs. 73% of UM, 5.45 pg/mL [3.9–7.9]; Supplemental Figure 2A). Correlation analyses of the various antibody profiles in the Ret^+^ CM group demonstrated antibodies associated with anti-phospholipid syndrome (APS) correlated with one another, namely anti-PL, anti-PS, and anti-CL (Supplemental Figure 3; Spearman’s rho [*R_s_*] range = 0.48–0.84, *P* < 0.005–0.0005). Anti–PS IgG levels correlated positively with anti-MPO and anti–PR3 IgG levels (PR3: *R_s_* = 0.48, *P* = 0.0008, MPO: *R_s_* = 0.53, *P* < 0.0002; Supplemental Figure 3) but not with anti-PF4/P levels. Anti-PF4/P antibodies did not correlate with the APS antibody panel. Anti-PF4/P IgG correlated with levels of antibodies against the neutrophil effector proteins anti–proteinase 3 (anti-PR3) (*R_s_* = 0.39, *P* = 0.005) and anti-MPO (*R_s_* = 0.51, *P* = 0.0002) (Supplemental Figure 3).

### Anti-PF4/P IgG levels are elevated in pediatric CM.

**Figure 2:**
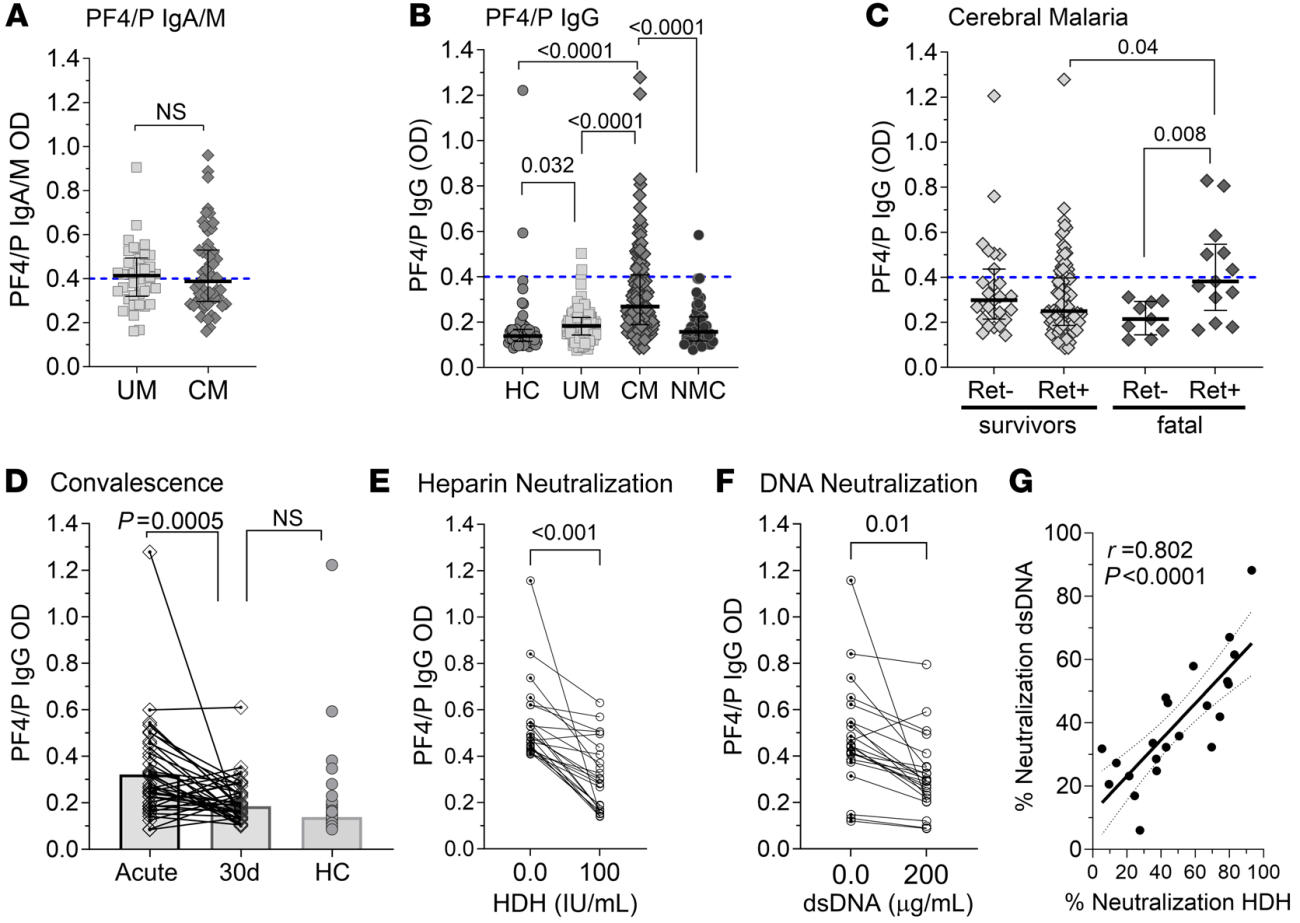
Anti–PF4/P IgG levels are elevated in pediatric CM. (**A**) Plasma levels of anti–PF4/P IgM/IgA antibodies in patients with UM (*n* = 38) versus patients with CM (*n* = 54). (**B**) Plasma levels of anti–PF4/P IgG in HCs (*n* = 56) versus UM (*n* = 124) versus CM (*n* = 136) versus NMC (*n* = 49) patients. (**C**) Plasma levels of anti–PF4/P IgG in CM survivors (Ret^–^ CM, *n* = 27; Ret^+^ CM, *n* = 87) and patients with fatal CM (Ret^–^ CM, *n* = 9; Ret^+^ CM *n* = 13). (**D**) Pair-wise comparison of anti–PF4/P IgG in acute versus convalescent plasma from patients with CM 30 days after admission (30d) (*n* = 39). (**E** and **F**) Pairwise analysis of neutralization of anti–PF4/P IgG binding in patient plasma with (**E**) 100 U/mL HDH (*n* = 24) or (**F**) 200 μg/mL dsDNA (*n* = 24). (**G**) Pearson’s correlation analysis between neutralization of anti-PF4/P binding by dsDNA versus HDH (*n* = 22). Shown within the graph is the Pearson’s rho coefficient (*r*) and the associated *P* value. In **A**–**C**, the assay cutoff threshold of OD above 0.4 is depicted by a dashed blue line. Shown are the median levels ± IQRs. Statistical significance was determined by Mann-Whitney *U* test (**A** and **D**), Kruskal-Wallis test with Dunn’s multiple comparisons (**B** and **C**), and a parametric paired *t* test (**E** and **F**).

We quantified the IgA/IgM (combined) isotypes of anti-PF4/P antibody levels in patient plasma and observed elevated levels above the cutoff (OD >0.4) of clinical significance (UM 52%, CM 48%) but saw no difference in median IgM/A levels between UM and CM patients (median OD: 0.414 vs. 0.388, *P* = 0.97) (Figure 2A). We focused our analysis on the IgG isotype, which is considered the clinically relevant isotype (26), and found that levels of anti–PF4/P IgG levels were elevated in patients with UM compared with healthy controls (HCs) (median OD = 0.139 [0.12–0.17]; *P* = 0.032) but did not differ significantly from the levels observed in NMC controls (median OD = 0.16 [0.12–0.22]; *P* = 0.52). Relative to both HC and NMC patient plasma, the levels of anti–PF4/P IgG in CM plasma were significantly elevated (CM vs. HC: *P* < 0.0001; CM vs. NMC: *P* < 0.0001) (Figure 2B).

When stratified by retinopathy status and outcome, we observed no difference in median PF4/P IgG levels between Ret^+^ CM survivors (median OD = 0.250 [0.19–0.43]) and Ret^–^ CM survivors (median OD = 0.298 [0.19–0.37]; *P* = 0.23). However, we observed elevated median levels of anti–PF4/P IgG in patients with fatal Ret^+^ CM (median OD = 0.381 [0.25–0.55] compared with both Ret^+^ CM survivors (median OD 0.25 [0.19–0.40]; *P* = 0.04) and patients with fatal Ret^–^ CM (median OD = 0.215 [0.14–0.29]; *P* = 0.008; Figure 2C). We quantified the levels of PF4/P IgG in available convalescent plasma of surviving CM patients (Figure 2D; 30 days [30d]). PF4/P IgG levels decreased with convalescence (acute CM: OD = 0.27 [0.19–0.41] vs. 30d convalescent: OD = 0.186 [0.14–0.24]; *P* = 0.0005). Anti–PF4/P IgG levels in convalescence were similar to those in HCs (HCs vs. CM 30d median OD: 0.16 vs. 0.18; *P* = 0.16; Figure 2D).

For a clinical HIT diagnosis, neutralization with high-dose heparin (HDH) (100 U/mL) is used as an additional verification of heparin-dependent anti-PF4/P antibody specificity (46). If neutralization does not occur, the result is considered equivocal, indicating that antibodies against a PF4/polyanion or PF4 antigen are present in the circulation, but their binding is not dependent on heparin or a polyanion for binding (47). When we tested neutralization of IgG binding to HIT antigen with HDH (100 U/mL), neutralization (determined as >50% inhibition) was significant relative to the control in 46% of samples (*P* < 0.0001; Figure 2E). Cell-free DNA (cfDNA), another naturally occurring polyanion (48), is elevated in malaria and associated with disease severity and survival status in children with CM (36). Since none of our patients received prophylactic heparin treatment, we tested and confirmed that DNA neutralized anti–PF4/P IgG binding (Figure 2F and Supplemental Figure 4) in a pattern similar to that of HDH (Pearson’s *r* = 0.80; *P* < 0.0001; Figure 2G).

### Anti-PF4/P antibodies are associated with markers of thromboinflammation.

**Figure 3:**
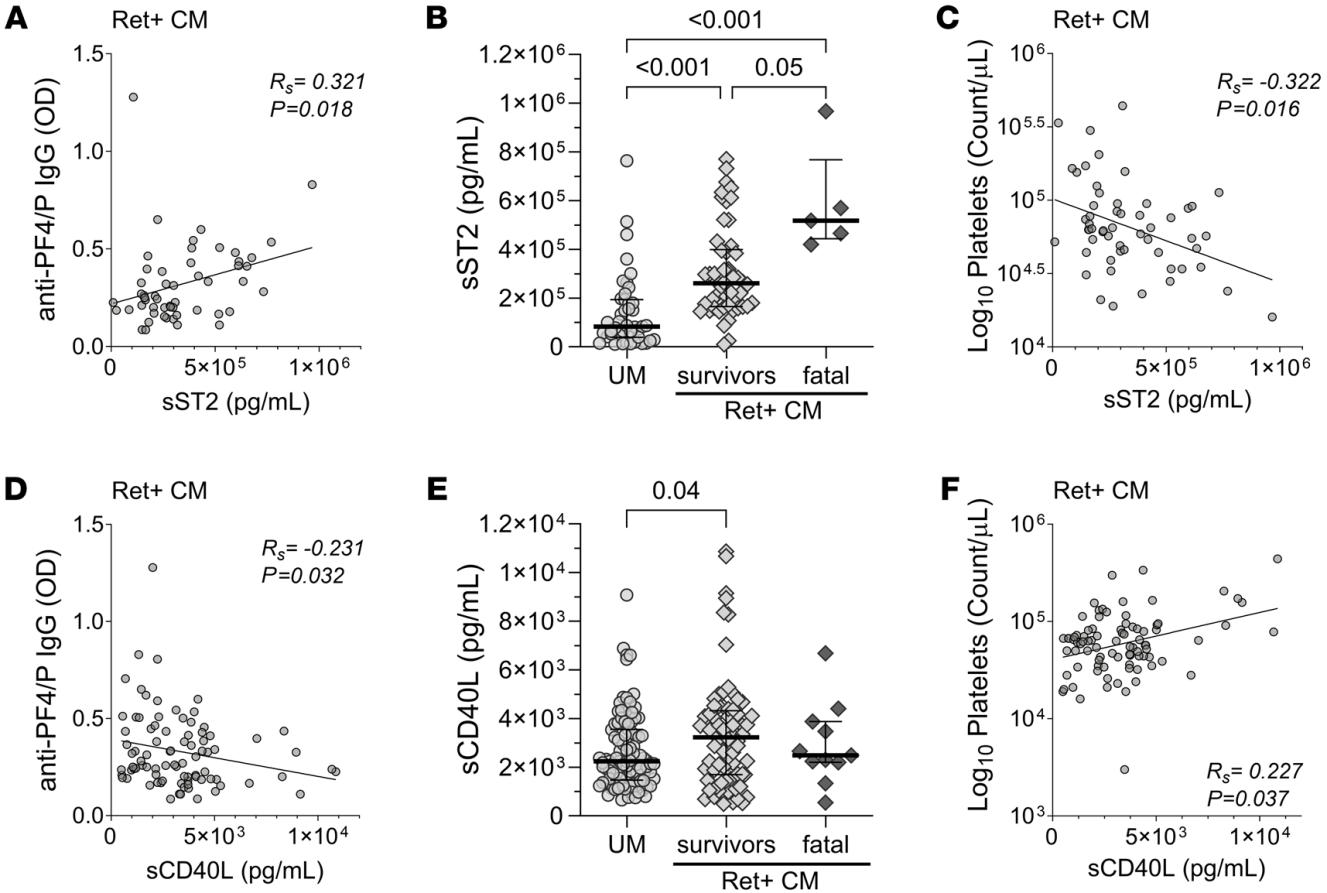
sST2 and CD40L levels, markers of thromboinflammation, link anti–PF4/P IgG levels in Ret^+^ CM with thrombocytopenia and disease outcome. (**A**) Spearman correlation analysis between anti–PF4/P IgG levels and sST2 plasma levels in patients with Ret^+^ CM (*n* = 54). (**B**) Plasma levels of sST2 in patients with UM (*n* = 43), Ret^+^ CM survivors (*n* = 50, and patients with fatal Ret^+^ CM (*n* = 5). (**C**) Spearman correlation analysis between peripheral platelet count and sST2 plasma levels in patients with Ret^+^ CM (*n* = 55). (**D**) Spearman correlation analysis between anti–PF4/P IgG levels and sCD40L plasma levels in patients with Ret^+^ CM (*n* = 86). (**E**) Plasma levels of sCD40L for patients with UM (*n* = 100), Ret^+^ CM survivors (*n* = 76), and patients with fatal Ret^+^ CM (*n* = 11). (**F**) Spearman correlation analysis between peripheral platelet count and sCD40L plasma levels in patients with Ret^+^ CM (*n* = 85). For **A**–**D** and **F**, the Spearman’s rho (*R_s_*) coefficient and associated *P* value are shown within the graphs. For **B** and **E**, the median with the IQR are shown for each sample set as a horizontal bar and error bars. Statistical significance in **B** and **E** was determined by Kruskal-Wallis test with Dunn’s multiple comparisons.

Markers of parasite burden such as peripheral blood parasitemia (Ret^+^ CM IgG: *R_s_* = 0.204, *P* = 0.043), *P*. *falciparum* cfDNA (*Pf* cfDNA) (UM IgG: *R_s_* = 0.323, *P* = 0.0002; Ret^+^ CM: *R_s_* = 0.286, *P* = 0.004), and total parasite load (*Pf* HRP2) (UM: *R_s_* = 0.317, *P* = 0.005) were positively correlated with anti–PF4/P IgG levels (Supplemental Table 1). Neutrophil activation and NET release are prothrombotic pathogenic processes that are associated with elevated levels of anti–PF4/P IgG in HIT (49–52). Markers of neutrophil activation and NETosis include MPO, a neutrophil effector molecule embedded within NETs, and cfDNA, a marker and degradation byproduct of neutrophil DNA release (53). In patients with UM, we observed a positive correlation of anti–PF4/P IgG with MPO (*R_s_* = 0.268, *P* = 0.032) and total cfDNA (*R_s_* = 0.248, *P* = 0.006) (Supplemental Table 1). Anti–PF4/P IgG correlated positively with the inflammation marker soluble suppressor of tumorigenicity 2 (sST2) in both UM (*R_s_* = 0.336, *P* = 0.028) and Ret^+^ CM (*R_s_* = 0.321, *P* = 0.018) (Supplemental Table 1 and Figure 3A).

**Figure 4:**
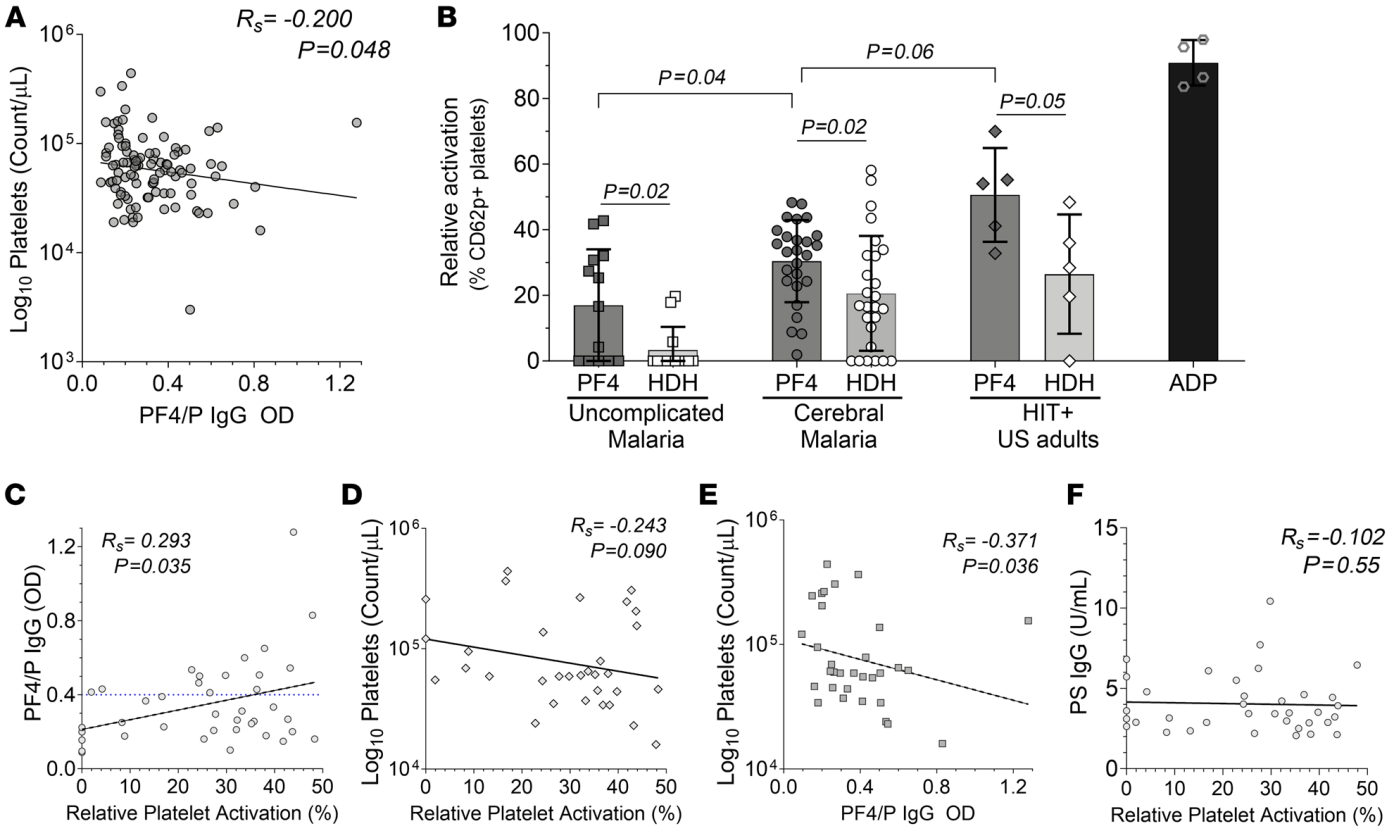
PF4/P IgG antibody levels in plasma from patients with CM are associated with decreased circulating platelets and platelet activation. (**A**) Spearman correlation analysis of circulating platelet levels in patients with Ret^+^ CM versus PF4/P IgG levels (*n* = 98) in plasma. Values were log transformed for linear regression analysis. (**B**) Heterologous platelet activation assay showing relative activation levels (percentage of CD62p/CD41) of donor platelets when incubated with plasma from patients with UM (*n* = 13) or CM (*n* = 26) or from HIT^+^ patient plasma (*n* = 5) in the presence of recombinant hPF4 (15 μg/mL, PF4) or recombinant hPF4 plus HDH (200 U/mL). Shown for each data point is the mean relative activation from 3 independent experiments. Treatment with ADP (10 μM) served as an internal positive control for maximal platelet activation. Statistical significance between hPF4-treated clinical groups was determined by Kruskal-Wallis test with Dunn’s multiple comparisons. Analysis within a clinical group for hPF4 treatment versus hPF4 plus HDH treatment was determined by Welch’s *t* test. Bar graph represents the mean ± SD. (**C**) Spearman correlation analysis of the relative platelet activation (*x* axis) in the subset of samples from **B**, plotted against the corresponding PF4/P IgG levels (*n* = 39) in patient plasma. (**D** and **F**) Spearman correlation analysis of the relative platelet activation (*x* axis) against (**D**) circulating platelet counts (*n* = 32) and (**F**) against anti–PS IgG levels (*n* = 36) in patient plasma. (**E**) Spearman correlation of PF4/P IgG plasma levels (*x* axis) plotted against circulating platelet counts (*n* = 32) for the subset of samples from **B**. Spearman’s *R_s_* coefficient, the associated *P* value, and calculated linear regression (dashed line) are shown within the graphs (**A** and **C**–**F**).

Among the markers associated with platelet activation and coagulation, we observed a positive correlation only in UM between anti-PF4/P and the marker for active coagulation, D-dimers (*R_s_* = 0.270, *P* = 0.039) (Supplemental Table 1) (7). In Ret^+^ CM, an inverse correlation was observed between anti–PF4/P IgG and soluble CD40 ligand (sCD40L), which is exposed to the platelet surface upon activation and subsequently shed (54) (*R_s_* = –0.231, *P* = 0.032; Figure 3D). Furthermore, we observed an inverse correlation between anti–PF4/P IgG and the circulating platelet count in Ret^+^ CM (*R_s_* = –0.201, *P* = 0.048; Supplemental Table 1 and Figure 4A). (55–57). Anti-PF4/P antibodies have not been described in association with anemia, but we observed an inverse correlation in patients with UM between anti-PF4/P and HgB (*R_s_* = –0.225, *P* = 0.015) and packed cell volume (PCV) (*R_s_* = –0.200, *P* = 0.029) (Supplemental Table 1).

Since the pathogenic function of HIT-like anti–PF4 IgG is primarily mediated through immune complexes that engage FcγRIIa receptors on platelets and monocytes (58, 59), we analyzed the associations of CICs in Ret^+^ CM with anti–PF4 IgG and soluble markers of thrombosis. We found that CIC levels were positively correlated with anti–PF4/P IgG in patients with Ret^+^ CM (*R_s_* = 0.252, *P* = 0.024; Supplemental Figure 2B and Supplemental Table 2). We observed a positive association between CIC and sST2 levels (*R_s_* = 0.326, *P* = 0.046), MPO levels (*R_s_* = 0.340, *P* = 0.002), sCD62p levels (*R_s_*= 0.445, *P* = 0.001), and *Pf* cfDNA levels (*R_s_* = 0.280, *P* = 0.009) in Ret^+^ CM. Similar to anti–PF4/P IgG, we also observed a negative correlation between CICs and markers of anemia (PCV: *R_s_* = –0.256, *P* = 0.040; HgB: *R_s_* = –0.313, *P* = 0.01) in patients with UM (Supplemental Table 2).

### Elevated levels of anti–PF4/P IgG in patients with Ret^+^ CM are associated with fatal outcome.

To better understand how sST2 and sCD40L plasma levels relate to anti–PF4/P IgG levels in CM, we plotted the values in relation to the following malaria case classifications: UM, Ret^+^ CM survival, or fatal Ret^+^ CM. The levels of sST2 were elevated according to malaria disease severity, with the highest median levels observed in patients with fatal Ret^+^ CM (517 × 10^3^ pg/mL [443 × 10^3^ to 768 × 10^3^]), followed by Ret^+^ CM survivors (260 × 10^3^ pg/mL [166 × 10^3^ to 399 × 10^3^]), and patients with UM (83 × 10^3^ pg/mL [39 × 10^3^ to 193 × 10^3^]) (Figure 3B). Moreover, sST2 levels in patients with Ret^+^ CM were negatively associated with circulating platelet counts (*R_s_* = –0.322, *P* = 0.016; Figure 3C). In a similar analysis, we observed that the median levels of sCD40L were higher in Ret^+^ CM survivors (3,228 pg/mL [1,695–4,323]) compared with patients with UM (2,239 pg/mL [1,465–3,548]; *P* = 0.05) (Figure 3E). The plasma levels of sCD40L in patients with fatal Ret^+^ CM (2486 pg/mL [2,202–3,879]) were lower than levels in survivors, although this observation was not statistically significant (Figure 3E). When we plotted sCD40L levels in Ret^+^ CM against the circulating platelet count, we observed a positive association (*R_s_* = 0.227, *P* = 0.037; Figure 3F), indicating that in Ret^+^ CM, lower levels of sCD40L in patient plasma were concomitant with thrombocytopenia. Logistic regression analysis of concurrent clinical diagnoses or outcome with anti–PF4/P IgG levels in Ret^+^ CM revealed a significant positive association with death as an outcome (OR = 15.2, 95% CI = 1.02–275; *P* = 0.048; Table 3), consistent with a role for anti-PF4/P in CM pathogenesis.

### PF4-dependent platelet activation induced by CM patient plasma is associated with anti–PF4/P IgG levels and thrombocytopenia.

In HIT, a follow-up platelet activation test confirms a positive diagnosis when anti–PF4 IgG levels exceed the clinical cutoff. Diagnostic evaluation assays used for HIT and vaccine-induced thrombotic thrombocytopenia (VITT) demonstrate the ability of patient plasma to activate normal platelets (60, 61). Relative to UM patient plasma, the level of platelet activation with CM plasma in the presence of PF4 was significantly elevated (UM: mean = 17% ± 17% vs. CM: mean = 30.4% ± 12.5%, *P* = 0.04) (Figure 4B). Platelets incubated with plasma from HIT^+^ patients (mean = 50.6% ± 14.2%) or the agonist adenosine diphosphate (ADP) (10 μM) served as positive controls. When HDH was added to neutralize the plasma-platelet interaction, we observed a decrease in the proportion of CD62p^+^ activated platelets for UM plus HDH (3.3% ± 7%; *P* = 0.02), CM plus HDH (20.6% ± 17.5%; *P* = .02), and HIT plus HDH (26.46% ± 18.2%; *P* = 0.05) (Figure 4B).

Platelet activation in the ex vivo functional assay was positively associated with PF4/P IgG levels in patient plasma (*R_s_* = 0.293, *P* = 0.035; Figure 4C) and trended in the direction of a negative correlation with the associated peripheral platelet count observed in the patients with malaria (*R_s_* = –0.243, *P* = 0.09; Figure 4D). In the subset of samples used in the ex vivo platelet activation assay, we also observed (as shown in Figure 4A) a negative association between the peripheral platelet count and the corresponding PF4/P IgG plasma levels (*R_s_* = –0.371, *P* = 0.036; Figure 4E).

The platelet activation observed when patient plasma was incubated with exogenous PF4 and normal platelets was not associated with anti–PS IgG levels (Figure 4F), indicating specificity of the platelet-activating properties induced by the patient plasma in the presence of exogenous PF4.

### Anti-PS antibodies are elevated in pediatric malaria infections.

**Figure 5:**
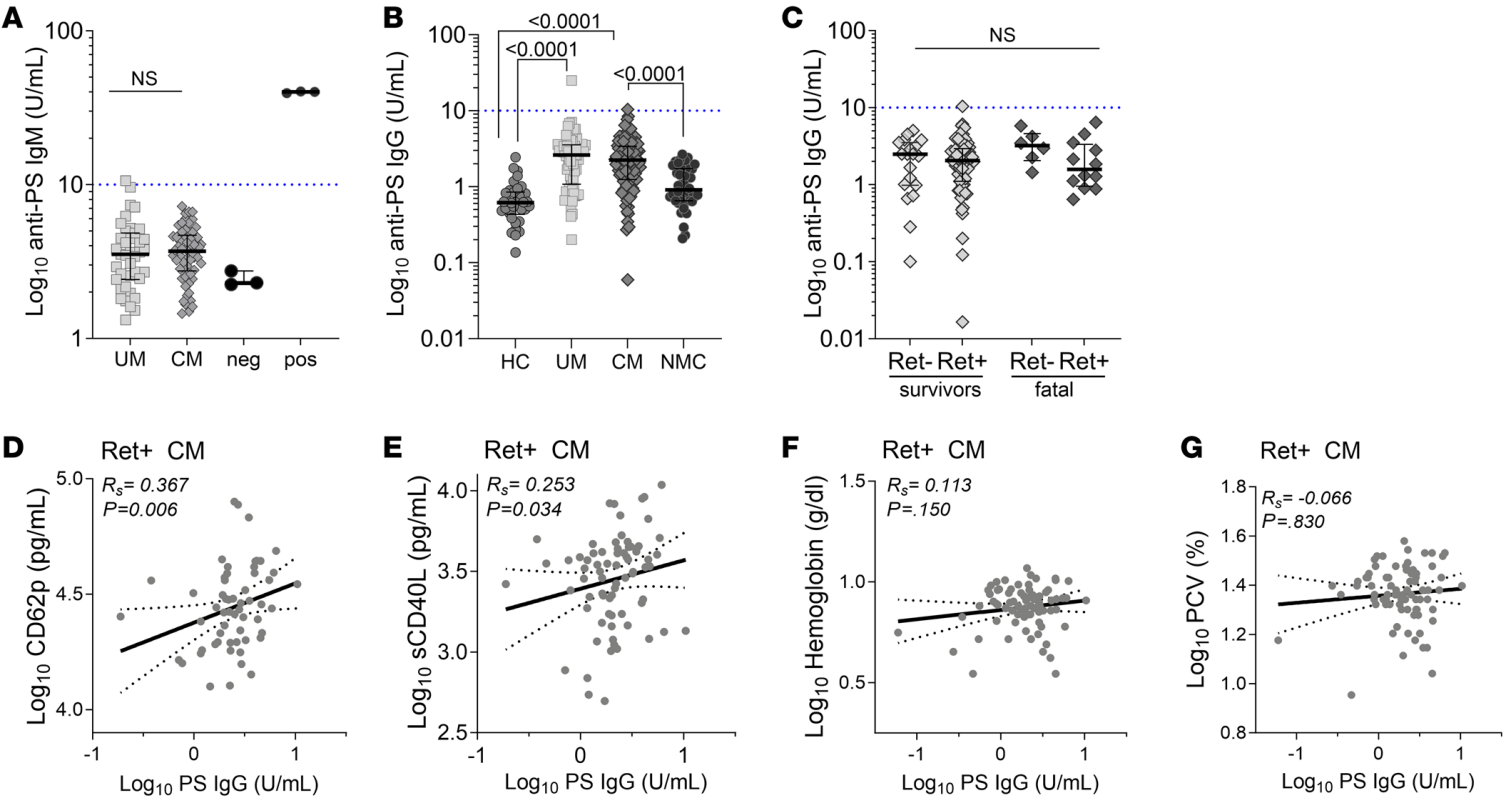
Anti-PS antibodies are elevated in malaria but do not vary with severity. (**A**) Plasma levels of anti–PS IgM antibodies measured in patients with UM (*n* = 39) versus patients with CM (*n* = 58). Shown are internal negative (neg) (*n* = 3) and positive (pos) (*n* = 3) controls. Dotted blue line across the *y* axis at 10 U/mL represents the predetermined assay clinical cutoff. (**B**) Plasma levels of anti–PS IgG in patients with UM (*n* = 74) versus patients with CM (*n* = 108) versus HCs (*n* = 34) versus patients with NMC (*n* = 48). (**C**) Plasma levels of anti–PS IgG in CM stratified by retinopathy status (Ret^–^ or Ret^+^) and outcome (survival or death). Ret^–^ survivors: *n* =19; Ret^+^ CM survivors: *n* = 63; Ret^–^ CM deaths: *n* = 6; and Ret^+^ CM deaths: *n* = 12. (**D**) CD62p versus anti–PS IgG (*n* = 58), (**E**) sCD40L versus anti–PS IgG (*n* = 73), (**F**) HgB versus anti–PS IgG (*n* = 77), and (**G**) PCV versus anti–PS IgG (*n* = 79). Shown within scatter plots are linear regression curves with the 95% CI in the dotted line, the calculated Spearman’s *R_s_*, and the associated *P* value. Shown are the median levels ± IQRs. Statistical significance was determined by Mann-Whitney *U* test (**A**) and Kruskal-Wallis test with Dunn’s multiple comparisons (**B** and **C**). Spearman correlation analysis of soluble markers in Ret^+^ CM patient plasma associated with platelet activation (**D** and **E**) or anemia (**F** and **G**) in relation to circulating levels of anti–PS IgG. Values were converted to log form to accommodate non-normal distribution.

We quantified anti–PS IgM levels and found no difference between median levels in UM and CM (Figure 5A). Comparing anti–PS IgG levels in UM and CM patients with HC and NMC controls, we observed that anti-PS levels were significantly elevated in patients with malaria infection compared with NMC controls for both UM and CM (HCs: 0.61 pg/mL [0.43–0.85] vs. UM: 2.6 pg/mL [1.2–2.2], *P* < 0.0001; NMC: 0.91 pg/mL [0.65–1.74] vs. UM: *P* < 0.0001; HCs vs. CM: 2.2 pg/mL [1.2–3.4], *P* < 0.0001; NMC vs. CM: *P* < 0.0001) (Figure 5B). We stratified the CM patient group by retinopathy status and fatal outcome but did not observe significant differences in anti-PS antibody levels in either comparison (*P* > 0.9999, Figure 5C).

A correlation analysis showed that anti–PS IgG was positively associated with markers of platelet activation including CD62p (UM: *R_s_* = 0.524, *P* < 0.0001; Ret^+^ CM: *R_s_* = 0.367, *P* = 0.006) and CD40L (UM: *R_s_* = 0.336, *P* = 0.005; Ret^+^ CM: *R_s_* = 0.253, *P* = 0.034) (Figure 5, D and E, and Supplemental Table 3). One proposed pathogenic prothrombotic function of anti-PS in diseases like APS and COVID-19 is through the activation of neutrophils, specifically the induction of NET release (NETosis) (22, 62). We observed a positive association between anti–PS IgG and MPO (*R*_s_ = 0.497, *P* = 0.005), host cfDNA (*R_s_* = 0.407, *P* = 0.017), and total cfDNA (*R_s_* = 0.405, *P* = 0.0004) in UM. In contrast, in Ret^+^ CM, host and total cfDNA levels correlated negatively with anti–PS IgG (host cfDNA: *R_s_* = –0.365, *P* = 0.001; total cfDNA: *R_s_* = –0.230, *P* = 0.048) (Supplemental Table 3).

Studies using malaria infection animal models and those involving *Plasmodium*-infected humans have reported an association of elevated levels of anti-PS with anemia through the direct targeting of anti-PS antibodies to the exposed PS lipid on erythrocytes during infection (37–39, 63, 64). We analyzed anti–PS IgG levels with markers of anemia and did not observe any correlations with PCV or HgB levels in either UM or Ret^+^ CM pediatric patients (Figure 5, F and G, and Supplemental Table 3). We also did not observe significant associations between anti–PS IgG levels in Ret^+^ CM pediatric patients who presented with a concurrent clinical complication of severe malarial anemia (SMA), respiratory distress (RD), jaundice, or with death as an outcome (Supplemental Table 4).

## Discussion

Autoimmune activity contributing to prothrombotic and inflammatory processes in the context of systemic infections has become increasingly appreciated (30, 65). Here, we evaluated whether prothrombotic autoimmune processes play a role in the pathogenesis of pediatric malaria. Of a panel of 8 autoantibodies, only levels of anti–PF4/P IgG, the primary pathologic agent of the clinical syndrome HIT (26), were elevated in the plasma of children with CM relative to those with UM. Compared with healthy pediatric community controls and sick comatose children (NMC controls) infected with non-*Plasmodium* infectious agents, anti–PF4 IgG levels were elevated in patients with malaria (UM and CM), suggesting that anti-PF4/P antibody production may be specific to *Plasmodium* infection. We observed a significant decline in anti-PF4/P antibody levels in convalescent patients, like what is observed in patients with HIT, VITT, or COVID-19, in whom antibody levels are transiently and acutely elevated under antigen exposure and inflammatory conditions, with a rapid decline in recovery (26).

Parallels in the clinical presentation between CM and other anti-PF4 disorders, such as HIT and VITT, include severe thrombocytopenia, endothelial dysfunction, evidence of consumptive coagulopathy, and microvascular thrombosis (7, 10, 26). We observed a positive association between anti–PF4/P IgG levels and markers of thromboinflammation including neutrophil activation and NETosis (i.e., cfDNA and the neutrophil effector MPO) and active coagulation (D-dimers) in patients with UM. Plasma from patients with CM had a greater capacity to activate normal platelets in vitro in a PF4-dependent manner compared with plasma from patients with UM. PF4-dependent (as opposed to heparin-dependent) platelet activation is typical in cases of autoimmune or spontaneous HIT such as VITT (31). Heparin-independent anti-PF4 antibodies, such as those of VITT, were shown to have greater binding strengths to PF4 molecules, which enhanced thrombus formation by immune complexes (66). Altogether, the data suggest that in UM, processes such as NETosis (MPO and cfDNA) and active coagulation (D-dimers) are early events in infection that correspond to elevated anti–PF4/P IgG levels.

In malaria, thrombocytopenia is associated with retinopathy, disease progression, and worse clinical outcomes (12, 67). In our analyses of patients with Ret^+^ CM, we observed a negative association between anti–PF4/P IgG levels and the peripheral platelet count and a positive association with mortality. We further showed that sST2 was elevated in patients with Ret^+^ CM, with the highest levels detected in patients with fatal Ret^+^ CM. ST2 levels were also positively associated with anti–PF4/P IgG levels and negatively associated with the peripheral platelet count. ST2 is an IL-1–like receptor for the alarmin IL-33, which is associated with neuroinflammation and vascular dysfunction (68–70), and was recently described as a prognostic marker of neurological sequelae in children with CM (55).

We also observed a negative association between plasma levels of anti–PF4/P IgG and sCD40L, an inflammatory immune modulator that bridges the adaptive and innate immunity and whose soluble levels are primarily derived from activated platelets (71). Interestingly, in patients with Ret^+^ CM, sCD40L levels, considered a marker for platelet activation, wane with reducing levels of peripheral platelets. Although not significant in our analysis, sCD40L median levels in patients with fatal Ret^+^ CM were reduced relative to those observed in Ret^+^ CM survivors, providing a potential link between a state of consumptive coagulopathy in patients with fatal Ret^+^ CM and pathogenic anti-PF4/P antibodies.

In HIT, anti-PF4/P antibody production is induced when PF4 tetramers released from platelets form complexes with heparin, a long polyanion, revealing a neoantigen on PF4 (72). PF4 molecules adhere to cell surfaces (58, 73) and interact with polyanions to form PF4/P antigen (74). Available polyanions include cfDNA from NETs or other damaged/necrotic cells (48, 49), heparan or chondroitin sulfate glycosaminoglycans on endothelial cells and monocytes (58, 75), procoagulant vWF strands secreted by endothelial cells (76), gram-negative bacterial cell wall components (77), and polyphosphates released by platelets (78). Cell sources of polyanions that can form HIT-like antigens are abundant in malaria (34–36, 79–83). As the endothelium is an active site of CM pathogenesis (84), it is likely that PF4/P antigen readily forms on the injured vascular wall(s) of children with CM, promoting immune complex aggregation and localized endothelial and immune cell activation. Additionally, during infection, platelets bind to iRBCs, deploying PF4, which accumulates and destroys the parasite within the erythrocyte (85–87).

Antibodies bound to PF4 can also form CICs with soluble polyanions such as cfDNA or vWF strands in the presence of anti-PF4/P antibodies. CIC levels were substantially elevated in approximately 75% of malaria cases tested here and positively correlated with anti-PF4/P, as well as with MPO, parasite cfDNA, sST2, and sCD62p in Ret^+^ CM. Thus, CICs could mediate pathogenic processes of anti-PF4/P immune complexes in patients with malaria through engagement of Fc receptors, such as FcγRIIA, or through opsonization.

Of the antibodies in the APS panel, we focused on anti–PS IgG, given the multiple independent reports of elevated levels in adult and pediatric cases of malaria caused by multiple infecting species including *P*. *vivax* and *P*. *falciparum* (37–39, 63, 88). The presence of anti-PS antibodies is also considered a risk factor for thrombosis and associated with disease severity in systemic lupus erythematosus (SLE), APS, COVID-19, and cardiovascular disease patients (89–95). PS is found on the inner leaflet of plasma membranes during basal conditions and is “flipped” to the outer leaflet of the plasma membrane under disease conditions to alert the system of stress or a breach (96). Major sources of PS include apoptotic or necrotic cells, neutrophils undergoing NETosis, and activated platelets (96, 97). In *Plasmodium* infection, PS is also found on microvesicles from iRBCs (98, 99), as well as on the surface of iRBCs and uninfected erythrocytes (39). Prior work characterizing the role of anti–PS IgG in anemia pathogenesis, both in vitro and in vivo, has focused on the destruction of PS-exposed, uninfected erythrocytes that in multiple independent studies and various patient cohorts have been associated with acute and post-acute malarial anemia (38, 39, 100, 101). In our pediatric CM cohort, we did not observe an inverse correlation of anti–PS IgG levels with markers of anemia (e.g., HgB and PCV) or a concurrent diagnosis of severe anemia, which is clinically defined as HgB nadir levels of 5 g/dL or lower. Although seemingly contradictory to previous studies, these results may indicate that different clinical presentations of severe malaria (i.e., SMA vs. CM) vary in their etiology and pathophysiology.

To date, no reports have linked anti-PS antibody function with thrombosis and/or procoagulant processes in pediatric CM. We observed positive associations between anti–PS IgG levels and markers of NETosis (e.g., MPO and cfDNA) in UM but not in Ret^+^ CM. We also observed a positive association between anti–PS IgG levels with sCD62p and CD40L, markers of platelet activation, in both UM and Ret^+^ CM patients, suggesting that elevated anti-PS antibodies may be involved in platelet activation. Unlike anti-PF4/P antibody levels, anti–PS IgG levels were not associated with CM, which supports a recent study of Ugandan pediatric patients with severe malaria, including CM (64). Furthermore, we observed an inverse correlation between anti–PS IgG and both cfDNA (host and total) and *Pf* HRP2, two markers strongly associated with worse outcomes in patients with Ret^+^ CM (12, 36, 102–104). Anti-PS antibodies could have a pathogenic or protective role depending on the disease context and the tissue microenvironment (105), just as NETs and neutrophils are important in host defenses but are also linked to pathogenic states including thromboinflammation. Antisera from patients with SLE containing anti-PS antibodies can be protective, recognize malaria antigens, and inhibit in vitro parasite growth (106). Further functional studies will be needed to explore the role, if any, of anti–PS IgG in prothrombotic processes in malaria.

Elevated levels of anti–PS IgG observed in malaria are produced by Tbet^+^ atypical memory B cell (atMBC) in response to IFN-γ and parasite DNA via TLR9, both of which are associated with malarial anemia (63, 101). Similarly, anti-PF4/P antibodies are produced from rapidly responding marginal zone B cells through TLR signaling in response to blood infections when antigen is presented via complement (107). The positive correlation we observed between *Pf* cfDNA and anti-PF4/P suggests another link between parasite-derived DNA and expansion of autoantibody-secreting B cells via TLR9. Whether Tbet^+^ atMBCs are a subset of marginal zone B cells that respond to parasite cfDNA via TLR9 to produce anti-PF4/P antibodies is not known. Rapidly responding, germinal center–independent Tbet^+^ B cells were tracked to the splenic marginal zone during viral infections (108), and a TLR9/IFN-γ–dependent expansion of Tbet^+^ B cells in the marginal zone of spleens was observed in a rodent model of malaria reinfection (109, 110). In-depth immunophenotyping of autoantibody-secreting B cells will be necessary to understand anti-PF4/P and anti-PS antibody production in pediatric malaria patients.

An inherent limitation in our analysis is the retrospective nature for all patient/case classifications. We were limited by a lack of longitudinal measurements of HgB and platelet counts. Studies evaluating the importance of anti-PS or anti-PF4/P antibodies have shown stronger correlations with nadir measurements of blood counts (31, 37, 38). We also did not have post-recovery clinical data to associate autoantibody levels with post-malaria anemia or post–acute thrombosis. An association of autoantibodies in post-recovery complications has been described for malaria (37, 39), as well as other infections, including post-recovery complications of COVID-19 (111–114).

Overall, our study points to a role for prothrombotic autoantibodies in pediatric CM. Induction of autoantibodies, such anti-PF4/P, in a subset of patients with malaria may be one of multiple mechanisms that tilts a UM case toward CM. Understanding the underlying pathologic immune processes of malaria thromboinflammation is imperative for the establishment of clinical interventions, generation of adjunct or prophylactic therapeutics, and improved patient prognoses. Our findings lay the groundwork for further investigations into autoimmune mechanisms contributing to thromboinflammatory processes in pediatric CM.

## Methods

### Sex as a biological variable.

The study design aimed to consent patients irrespective of sex, such that the patient recruitment would be unaffected by selection biases toward 1 sex or the other. Our study examined both male and female pediatric patients, and our analysis of key variables presented here did not find differences with regard to sex.

### Patient cohort and sample collection.

Patients with UM (*n* = 124) were recruited during consecutive malaria seasons (January–June) in 2016–2017 and met the following criteria: children aged 1–12 years old, who presented to the Accident and Emergency (A&E) Department at Queen Elizabeth Central Hospital (QECH) in Blantyre, Malawi, with a history of fever, a positive smear score for peripheral *P*. *falciparum* parasitemia as calculated by thick blood smears (parasites/100 fields) (115), consciousness, and no overt signs of compromised health, malnutrition, or progression to severe malaria. Patients with UM were treated as outpatients within the A&E Department of QECH and medicated according to national protocols (116). Patients with CM (*n* = 136) were recruited during consecutive malaria seasons (January–June) in 2015–2017 (Figure 1 and Table 1). Children aged 6 months to 12 years were admitted to the Pediatric Malaria Research Ward at QECH and met the criteria for WHO-defined CM (i.e., *P*. *falciparum* parasitemia, coma that was not resolved despite treatment for hypoglycemia and/or seizures, and exclusion of other identifiable causes of coma). The BCS, a clinical assessment on a scale of 0 (no consciousness) to 5 (consciousness), was used to determine the level of consciousness with scores of 2 or lower indicating a comatose state (103, 117). Retinal fundoscopy was performed by a trained ophthalmologist and stratified the CM patients into either Ret^–^ CM (*n* = 36; i.e., normal ocular fundi) or Ret^+^ CM (*n* = 100; i.e., observation of retinal whitening, hemorrhages, and/or abnormal vessels). Children who recovered from CM (*n* = 39) were reassessed 30 days after admission, and convalescent plasma was collected for analysis.

HCs (*n* = 56) aged 7 months to 11 years were recruited from Ndirande Health Centre (Figure 1 and Table 1) during well checkups. They presented with no symptoms and were negative for *Plasmodium* infection by thick blood smears. Children with NMC (*n* = 49) were admitted to the QECH Pediatric Research Ward as part of an ongoing prospective study (CHildhood Aetiologies of Severe Encephalopathy [CHASE]) (Figure 1 and Table 1). Patients with NMC were recruited between February 2018 and April 2020 and included children aged 3 months to 14 years who were negative for *P*. *falciparum* infection by microscopy and rapid diagnostic test. They presented with fever, a deep coma (BCS ≤2), and a suspected CNS infection not of malarial etiology.

For UM, CM, NMC, and HC participants, 4 mL of blood was drawn into citrate anticoagulant tubes and 0.5 mL blood was drawn into EDTA tubes at the time of study enrollment. The number of samples used in each assay is reported in the text, figures, or figure legends. For some assays, 4 mL citrated plasma from adult volunteers or patients in the United States was collected following IRB-approved protocols.

### Autoantibody ELISA analysis.

Plasma or serum samples were analyzed for the presence of anti-PR3, anti-MPO, anti-PS, anti-CL, anti-PL, and anti–β-2-glycoprotein I (anti-β2GPI) IgG antibodies using commercially available ELISA kits (Orgentec Diagnostika) designed for in vitro diagnostic use. Anti–PS IgM antibody levels were also measured. Briefly, samples were diluted 1:100 in diluent buffer per the manufacturer’s protocol and then detected with an HRP-conjugated anti–human IgG secondary antibody followed by incubation with 3,3′, 5,5′-tetramethylbenzidine (TMB) chromogenic substrate. Plasma antibody concentrations (U/mL) were determined using OD and a standard curve. Levels were considered clinically relevant and positive at predetermined recommended cutoff levels of 10 U/mL or higher for anti-CL, anti-PL, anti-PS, and anti-β2GPI and of 5 U/mL or higher for anti-PR3 and anti-MPO.

Levels of complement protein C1q-associated CICs (CIC-C1q) were quantified in plasma using MicroVue’s CIC-C1q enzyme immunoassay (EIA) according to the manufacturer’s instructions (Quidel Corp.). CIC levels were determined with a validated standard curve and a clinical cutoff of 4 μg/mL per the kit specifications. Control samples were added to all plates for reference and included pooled plasma from SLE patients (118) and plasma from healthy adult American volunteers.

Total anti–dsDNA IgG was measured using a modified ELISA protocol from a published protocol (118). Briefly, plates were coated overnight with salmon sperm DNA (Thermo Fisher Scientific). Plasma from each participant was added to the plate at a 1:50 dilution and incubated for 2 hours at room temperature. Alkaline phosphatase–conjugated goat anti–human IgG (Southern Biotech) was added for 1 hour and developed with a phosphatase substrate (MilliporeSigma). Positive and negative controls were the same as those described for the for CIC-C1q EIA. All UM, CM, SLE, and HC ODs were normalized to the average of 2 SLE patients with known high levels of anti-dsDNA antibody. The cutoff was determined by the mean plus 3 SDs of the HC samples.

Antibody (IgG or IgM/A) levels specific to PF4 complexed to polyanions (PF4/P) in patient samples were determined using a clinical diagnostic immunosorbent assay for HIT (PF4 Enhanced Assay, Immucor). Positive tests were defined as samples with an OD of 0.400 or higher according to the manufacturer’s predetermined cutoff. To determine polyanion neutralization, a high dose (100 U/mL) of unfractionated high-molecular-weight heparin (HDH) or 200 μg/mL dsDNA was added to each diluted plasma sample prior to incubation on a coated ELISA plate. The neutralization percentage was determined as described in the manufacturer’s protocol and calculated as follows: [1 – (OD sample w/ polyanion)/(OD sample alone) × 100].

All ELISA and EIA assays used in this study, internal positive and negative controls, and standard curve calibrators were validated with OD values that complied with kit specifications. Measurements were made on a Molecular Devices SpectraMax M2 (USF) or a Biotek ELx800 (University of Malawi) plate reader, and values were converted to U/mL based on a 4-parameter logistic regression (4PL) analysis (GraphPad Prism 9).

### Soluble thrombosis marker quantification.

Plasma IL-8 levels were measured using Cytokine Bead Arrays (BD Biosciences). The data obtained from this analysis were partially previously published (36, 119). Briefly, plasma samples were incubated with capture beads and processed according to the manufacturer’s protocol. Data were acquired on a CyAn ADP flow cytometer (Beckman Coulter) and analyzed with BD FCAP software, version 3.0 (BD Biosciences).

D-dimers, sST2, P-selectin (CD62p), and cluster of differentiation 40 ligand (CD40L) were analyzed using a custom magnetic bead–based Luminex assay (R&D Systems Luminex Assays). Diluted plasma (1:2) was incubated with Luminex beads overnight (18 hours) at 4°C and processed by washing and subsequently incubating with biotin-conjugated antibody and streptavidin as stipulated by the kit protocol. Bead counts were acquired on a MagPix system (Luminex), and concentrations were calculated using Luminex xPONENT software.

Plasma concentrations of MPO were measured by commercial ELISA (Human MPO ELISA kit, R&D Systems). Sample dilutions were modified (manufacturer-suggested, 1:10) to a 1:50 dilution to allow for a range of readings within the standard curve. OD measurements were taken at 450 nm using a Molecular Devices SpectraMax M2 (USF) or a Biotek ELx800 (University of Malawi) plate reader, and values were converted to pg/mL based on the corresponding standard curve.

Total cfDNA levels were measured in plasma samples by commercial fluorometer (Qubit 4 Fluorometer; Qubit dsDNA Quantification Assay, Invitrogen, Thermo Fisher Scientific) as previously described (36). The levels of host- or parasite-derived cfDNA were determined with a probe-based quantitative PCR method as previously described (36).

*Pf* HRP2 was measured by ELISA according to validated methods (102) using a commercial kit (Cellabs, Brookvale, Australia).

### Ex vivo platelet activation by P-selectin expression assay.

Platelet activation was assessed using a modified P-selectin expression assay (PEA) based on previously published methods (60, 61). Blood from healthy adult volunteers was collected in sodium citrate tubes and processed to collect platelet-rich plasma (PRP) by low-force centrifugation at 100*g* for 5 minutes at room temperature. PRP (3 μL) from 2–3 donors was incubated with 10 μL patient plasma in the presence of human PF4 (hPF4) (15 μg/mL) with or without HDH (200 U/mL), or saline only (no treatment) in a total activation volume of 20 μL. PRP samples were incubated for 60 minutes at room temperature. Samples were then stained with anti–human CD41-APC (clone P2, Beckman Coulter) and anti–human CD62p-PE (clone CLBThromb/6, Beckman Coulter) for 20 minutes, fixed with 1% paraformaldehyde for 10 minutes at room temperature, and analyzed by flow cytometry (Beckman Coulter CytoFLEX). The positive control for maximum activation was treated with PRP with 10 μM ADP for 8 minutes prior to staining with antibodies and fixation with 1% PFA, as described above. PRP incubated with normal platelet-poor plasma (PPP) served as the negative control and was used to normalize sample values. Platelets were identified by forward and side scatter and gated by CD41^+^ staining. CD41^+^ platelets were then analyzed by histogram for CD62p staining (Supplemental Figure 5). The following equation determined the fold-change reactivity to PF4 treatment on platelets incubated with patient plasma: percentage of relative platelet activation = [(PF4-treated – no treatment)/(PF4-treated)] × 100.

### Statistics.

Descriptive and univariate analyses were performed in GraphPad Prism 9 (GraphPad Software)) or SPSS 28 (IBM). Mann-Whitney *U* nonparametric analysis and a parametric *t* test with Welch’s correction were used for unpaired comparisons. The Wilcoxon signed-rank test was used for paired data. Kruskal-Wallis with Dunn’s post hoc test was used for multiple comparisons. The resultant data are presented as the median ± IQR or Spearman’s rho (*R*_s_). To adjust for multiple comparisons when performing a correlation matrix analysis, the 2-stage step-up method of Benjamini, Krieger, and Yekutieli (120) with a *Q* value set to 1 was carried out. The χ^2^ test was performed for categorical data analyses, which are presented as *n* (percentage of group) values. Spearman’s correlation analyses were conducted for associations between continuous variables. Logistic regression was used to identify associations with binary variables, such as clinical risk factors for SMA, RD, lactic acidosis, jaundice, and death. All statistical analyses were 2 tailed, and *P* values of 0.05 or less were considered statistically significant.

### Study approval.

These studies were approved by the IRBs at the University of Malawi College of Medicine, the University of South Florida, and Michigan State University. Informed consent was obtained from the parent or legal guardian of all pediatric study participants or directly from the study participant (American healthy adult volunteers or patients) prior to enrollment. Our study involved both male and female children, and similar findings are reported for both sexes.

### Data availability.

The underlying values for plotted graphs within the main text and the supplemental material are available in the Supporting Data Values file.

## Author contributions

KK, AK, MRG, and IMV conceptualized the study. IMV, AK, and VH performed experiments. Data were analyzed by IMV, and TEK performed and advised on statistical regression analyses. AK and VH collected and processed the CM and UM patient samples for the Blantyre Malaria Project (BMP). STJR and WLM supervised the recruitment of patients with UM. AA processed patient samples for CHASE. KBS and TET recruited patients, provided clinical data for patients with CM, and provided intellectual input. STJR recruited patients and provided clinical data for CHASE. IMV and AK wrote the manuscript with guidance from the corresponding authors (KK and KBS) and edits and input from all authors.

## Supplementary Material

Supplemental data

ICMJE disclosure forms

Supporting data values

## Figures and Tables

**Table 3:**
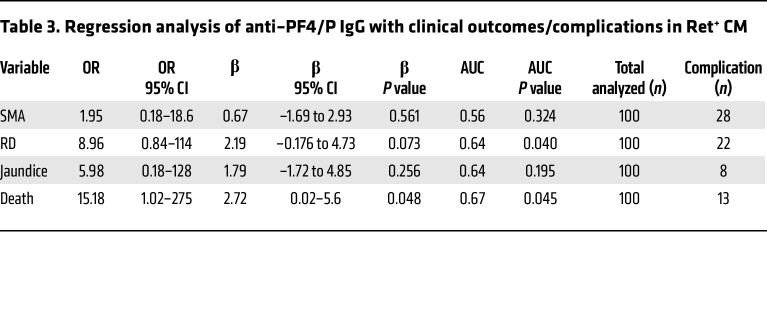
Regression analysis of anti–PF4/P IgG with clinical outcomes/complications in Ret^+^ CM

**Table 2:**
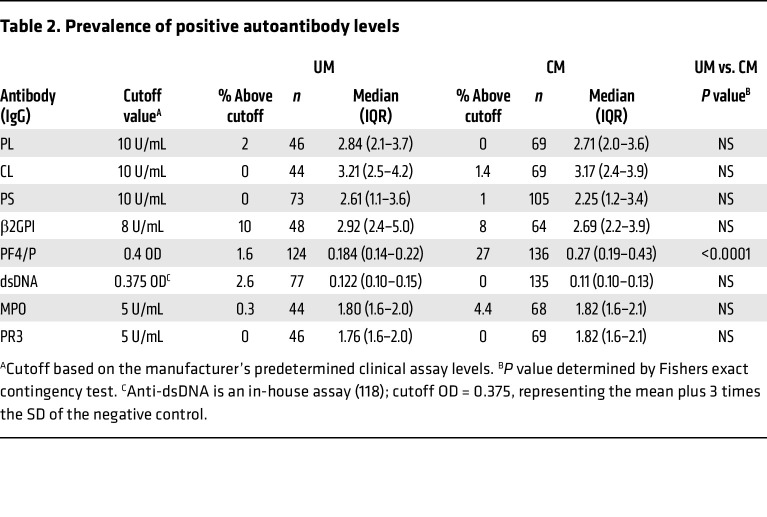
Prevalence of positive autoantibody levels

**Table 1:**
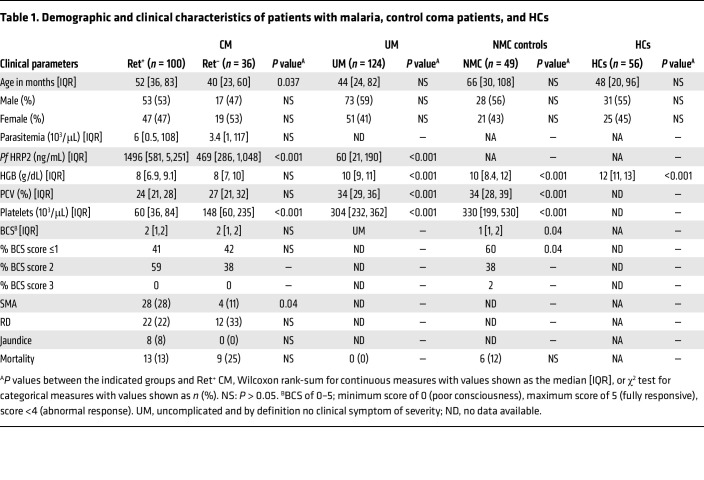
Demographic and clinical characteristics of patients with malaria, control coma patients, and HCs

## Version 1 04/23/2024

In-Press Preview

## Version 2 06/03/2024

Electronic publication
