# Supplementary material for: Prothrombotic autoantibodies targeting platelet factor 4/polyanion are associated with pediatric cerebral malaria
Source: J Clin Invest. 2024 Apr 23;134(11):e176466. doi: 10.1172/JCI176466 (PMC11142751; doi:10.1172/JCI176466)
Supplement: Supplemental data [file jci-134-176466-s221.pdf]

Vera 2023_Supplemental Figures

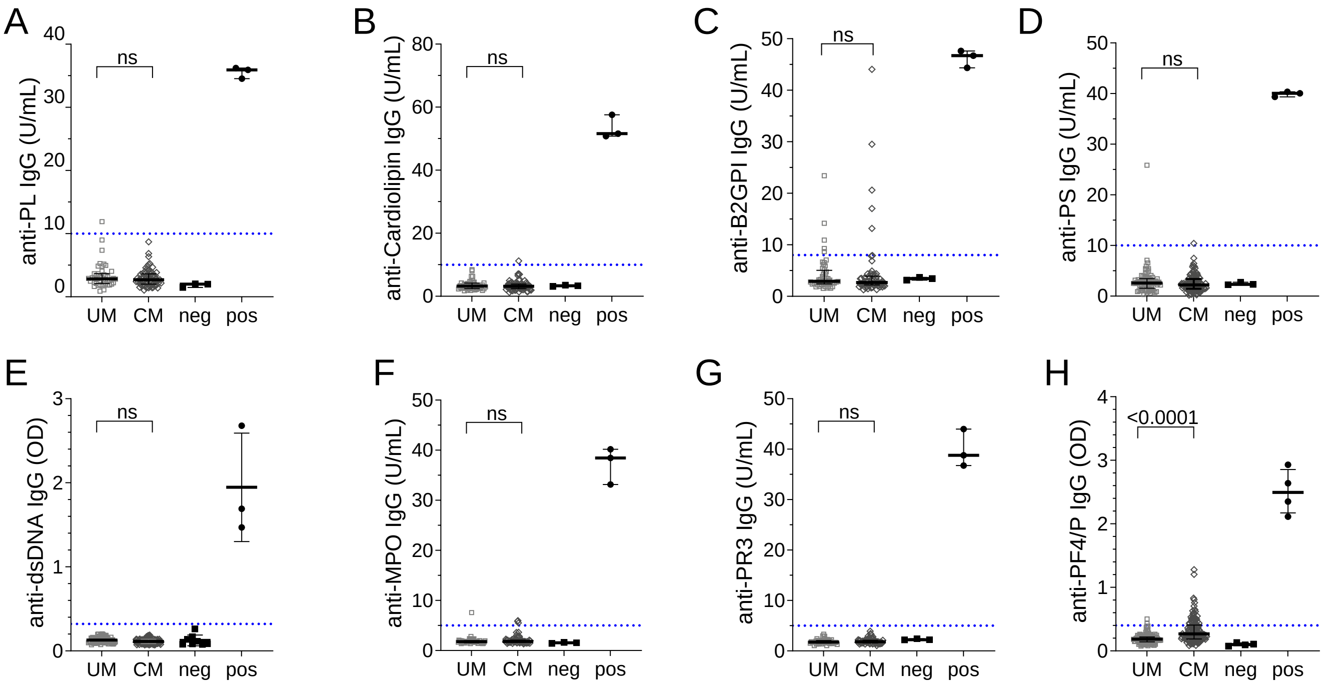

Supplemental Figure S1. Analysis of procoagulant autoantibodies in uncomplicated (UM) versus cerebral malaria (CM).

Patient plasma samples (UM and CM) were analyzed for detection of autoantibodies A) anti-Phospholipid, (anti-PL; UM n=46, CM n=69), B) anti-Cardiolipin (UM n=44, CM n=69), C) anti β2-Glycoprotein-I (anti-β2GPI; UM n=48, CM n=64), D) anti-Phosphatidylserine (anti-PS; UM n=74, CM n=105), E) anti-dsDNA (UM n=77, CM n=135), F), anti-Myeloperoxidase (anti-MPO; UM n=44, CM n=68), G) anti-Proteinase 3 (anti-PR3; UM n=46, CM n=69), and H) anti-Platelet Factor 4/Polyanion (anti-PF4/P; UM n=124, CM n=136). Data for UM and CM in (D) and (H) is also included in main text on Figures 5B and 2B, respectively. Interpolated antibody concentrations for A-D and F-G are shown as Units per milliliter (U/mL). For E and H, optical density values (OD) are shown. Dashed blue line represents the clinical cutoff based on the diagnostic assay, except for anti-DNA (E) which was an in-house assay and cutoff was determined as mean of negative control + 3x standard deviation. Internal negative controls (neg, n=3-4) and positive controls (pos, n=3-4) are shown. For anti-DNA, controls are plasma from healthy American adult volunteers (neg) and American SLE patients (pos). Mann-Whitney non-parametric analysis was used to determine statistical significance between UM and CM.

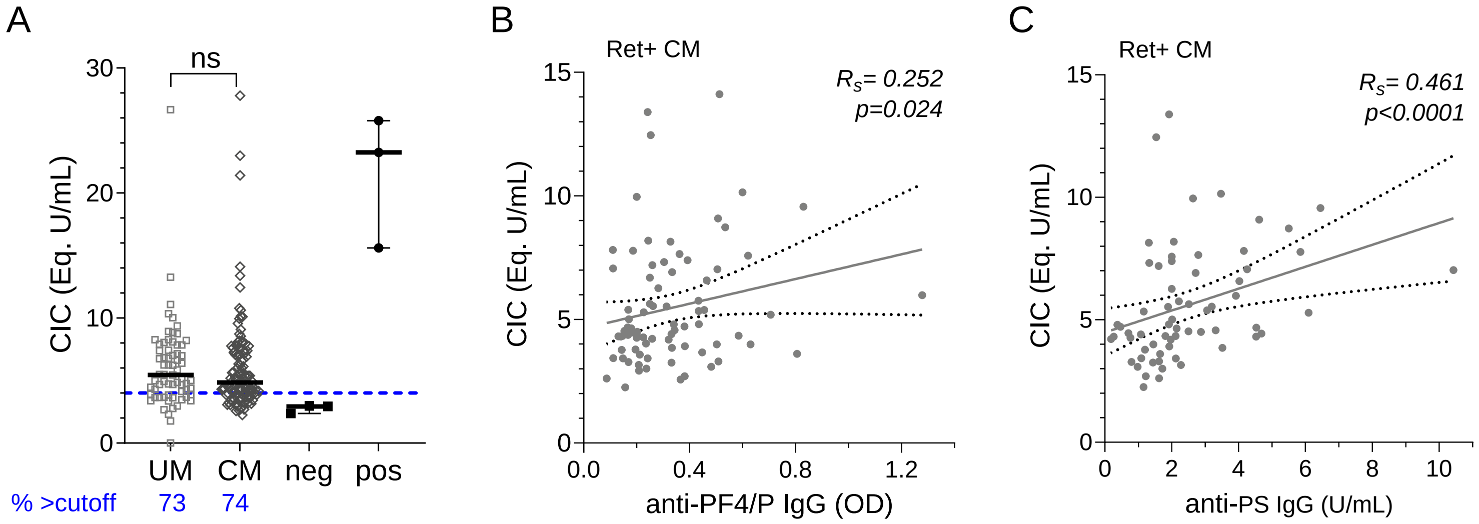

Supplemental Figure S2. Circulating immune complexes (CIC) are elevated in malaria.

A) Patient plasma samples for uncomplicated malaria (UM, N=67) and cerebral malaria (CM, N=121) were analyzed via ELISA assay for detection of circulating immune complexes (CIC). Calculated concentrations for CIC are shown as equivalent units per milliliter (Eq. U/mL)/mL). Comparisons between UM and CM were analyzed by Mann-Whitney non-parametric analysis. Dashed blue line represents the diagnostic assay's pre-determined cutoff level at 8 Eq. U/mL. Percent prevalence of patient samples above the cutoff (% >cutoff) are shown below x-axis label. Controls, included for reference, represent healthy American adult volunteers (neg, N=3) and American SLE patient plasma (pos, N=3). Spearman correlation analysis of CIC in Ret+ CM patient samples with levels of B) anti-PF4/P IgG, and (C) anti-PS IgG. Spearman rho (R_s) and p-value (p) are shown within graph.

Spearman correlation Matrix - Autoantibodies in UM

	anti-PF4/P	anti-PR3	anti-MPO	anti-PL	anti-PS	anti-B2GPI	anti-CL	anti-dsDNA
anti-PF4/P	1.00							
anti-PR3	0.11	1.00						
anti-MPO	0.40	0.43	1.00					
anti-PL	0.14	0.04	0.19	1.00				
anti-PS	-0.26	0.23	0.01	0.61	1.00			
anti-B2GPI	0.06	-0.04	0.18	0.56	0.08	1.00		
anti-CL	0.22	-0.01	0.25	0.61	0.67	0.32	1.00	
anti-dsDNA	0.04	0.18	0.20	0.29	0.45	-0.02	0.50	1.00

P-value*
ns
<0.0001
<0.0012

**BKY discovery cutoff p-value = 0.0012*

N=14-78

Spearman correlation Matrix - Autoantibodies in Ret- CM

	anti-PF4/P	anti-PR3	anti-MPO	anti-PL	anti-PS	anti-B2GPI	anti-CL	anti-dsDNA
anti-PF4/P	1.00							
anti-PR3	0.16	1.00						
anti-MPO	0.40	0.65	1.00					
anti-PL	0.11	0.41	0.33	1.00				
anti-PS	-0.13	0.46	0.46	0.76	1.00			
anti-B2GPI	0.36	-0.30	-0.18	0.11	0.30	1.00		
anti-CL	0.51	0.38	0.22	0.72	0.85	0.45	1.00	
anti-dsDNA	0.09	-0.03	-0.13	0.09	0.08	0.72	0.37	1.00

P-value
ns
<0.0001
<0.0016

**BKY discovery cutoff p-value = 0.0016*

N=18-38

Spearman correlation Matrix - Autoantibodies in Ret+ CM

	anti-PF4/P	anti-PR3	anti-MPO	anti-PL	anti-PS	anti-B2GPI	anti-CL	anti-dsDNA
anti-PF4/P	1.00							
anti-PR3	0.39	1.00						
anti-MPO	0.51	0.52	1.00					
anti-PL	0.15	0.33	0.46	1.00				
anti-PS	0.16	0.48	0.53	0.84	1.00			
anti-B2GPI	0.08	-0.06	0.16	0.35	0.11	1.00		
anti-CL	0.22	0.47	0.42	0.65	0.75	0.14	1.00	
anti-dsDNA	0.03	0.38	0.24	0.30	0.25	0.16	0.34	1.00

P-value
ns
<0.0001
<0.001
<0.0057

**BKY discovery cutoff p-value = 0.0057*

N = 43 - 100

Supplemental Figure S3. Inter-correlation of autoantibodies in plasma of malaria patients.

Spearman correlation analysis of autoantibody levels versus one another for UM, Ret- CM and Ret+ CM samples. Value within each cell of the heatmap is the calculated Spearman Rho. Each cell is color coded based on p-value significance as denoted in the legend. Multiple comparisons were adjusted using Benjamini, Krieger and Yekutieli Significance p-stack analysis method. Only values meeting the adjusted BKY p-value threshold for discovery are color coded in the heatmap and considered significant.

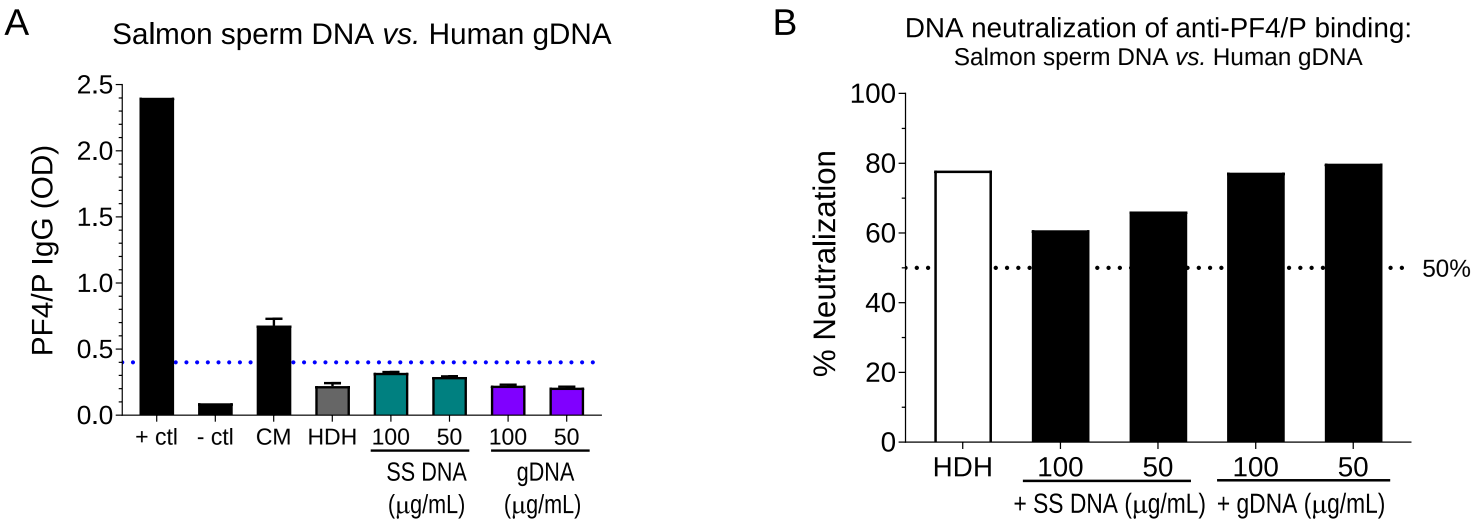

Supplemental Figure S4. Double stranded DNA neutralization of IgG antibody binding to PF4:polyanion antigen.

A) Patient plasma sample (CM) with elevated anti-PF4/P IgG was tested in EIA assay for PF4/P binding in the presence of double stranded DNA (SS; Salmon Sperm DNA) or extracted human genomic DNA (gDNA) at 50 or 100μg/mL. High dose heparin (HDH) at 100U/mL was included as control of inhibition. Positive (+ ctl) and negative (- ctl) internal assay controls are included for reference. Optical density (OD) values are plotted for anti-PF4/P binding. OD value ≥ 0.40 cutoff is marked by the dashed blue line across the y-axis. Shown is mean ± standard deviation. (B) Data from (A) converted to % neutralization calculated as [1-(OD sample w/ polyanion) ÷ (OD sample alone) × 100]. Neutralization of antibody binding that is greater than 50% (depicted by the black dashed line across the y-axis) is considered a positive test result. Data shown is representative of n=2 experimental replicates.

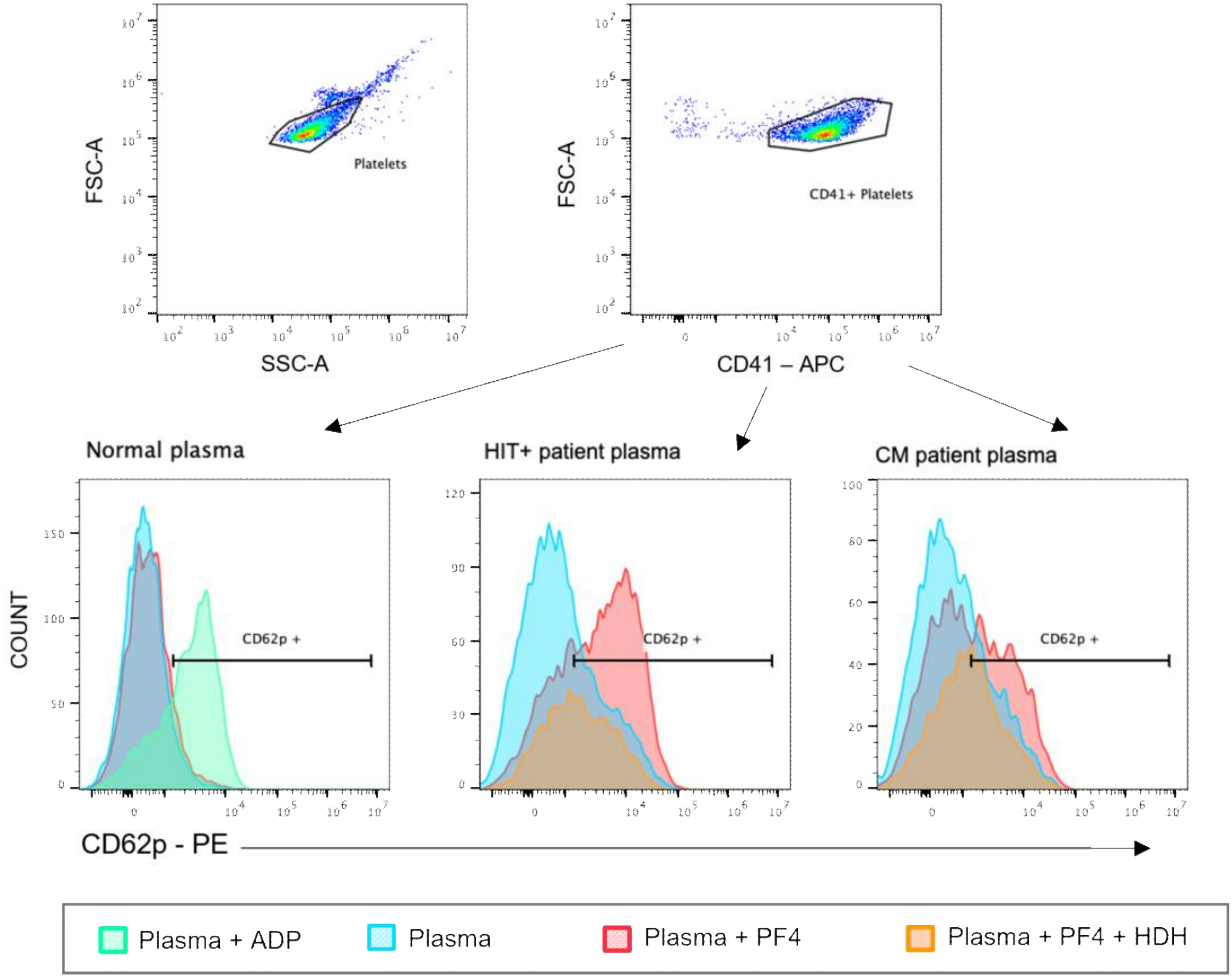

Supplemental Figure S5. Flow cytometry detection of patient plasma-activated platelets in ex-vivo heterologous assay.

Gating strategy for detection of activated platelets that express surface CD62p activation marker. Platelets within platelet rich plasma (prp) were identified by FSC/SSC in log scale and discriminated by staining with the pan-platelet specific marker CD41-APC. Platelet activation was measured by detection of anti-CD62p-PE and a shift to the right on the histogram within the CD41+ population. Normal Plasma is from healthy control volunteers serves as a negative control (blue histography), Normal plasma that was stimulated with 10µM adenosine diphosphate (ADP; green histograph) serves as an internal positive control. Platelet stimulation with plasma from HIT+ patient or CM patient plasma under added exogenous hPF4 (15µg/mL) is shown in red histography. HIT+ or CM patient plasma with added exogenous hPF4 (15µg/mL) + high dose heparin (HDH, 200U/mL) to neutralize activation is shown in orange histograph.

Supplemental Table S1.

Correlation of immune and clinical markers associated with anti-PF4/P IgG.

Process	Variable	UM			Ret+ CM		
		R_s	p-value	N	R_s	p-value	N
NETosis, Inflammation & Cell Damage	Total cfDNA	**0.248**	**0.006**	**123**	0.170	0.091	100
	Host cfDNA	0.223	0.0530	76	0.178	0.079	99
	sST2	**0.336**	**0.028**	**43**	**0.321**	**0.018**	**54**
	MPO	**0.268**	**0.032**	**64**	0.156	ns	98
	IL-8	0.111	ns	117	0.201	0.068	83
Coagulation & Platelet activation	D-dimers	**0.270**	**0.039**	**59**	0.165	ns	46
	sCD62p	-0.102	ns	95	0.114	ns	71
	sCD40L	-0.060	ns	58	***-0.231***	***0.032***	***86***
Parasite burden	PfHRP2	**0.317**	**0.007**	**71**	0.171	0.089	100
	Pf cfDNA	**0.323**	**0.0002**	**123**	**0.286**	**0.004**	**99**
	Parasitemia	ND	-	-	**0.204**	**0.043**	**99**
Clinical parameter	PCV	**-0.200**	**0.029**	**119**	-0.140	ns	100
	Hemoglobin	**-0.225**	**0.015**	**117**	-0.124	ns	97
	Platelets	0.115	ns	76	**-0.201**	**0.048**	**98**

UM: Uncomplicated Malaria; Ret+ CM: Retinopathy positive cerebral malaria;Rs: Spearman Rho; cfDNA: cell free DNA; sST2: soluble Suppression of Tumorigenicity 2; MPO: Myeloperoxidase; IL-8: Interleukin 8; PfHRP2: P. falciparum Histidine Rich Protein 2; PCV: Packed Cell Volume.Variables with a significant p-value < 0.05 are bolded and italiziced. P-values ≤ 0.05 are bolded; ND: Not determined; ns: not significant.

Supplemental Table S2.

Immune and clinical markers associated with Circulating Immune Complexes.

Process	Variable	UM			Ret+ CM		
		R_s	p-value	N	R_s	p-value	N
Autoantibody	anti-PF4/P IgG	**0.325**	**0.007**	**67**	**0.252**	**0.024**	**79**
	anti-PS IgG	**0.438**	**0.014**	**31**	**0.461**	**<0.001**	**64**
NETosis & Cell Damage	Total cfDNA	0.160	ns	66	0.167	ns	86
	Host cfDNA	-0.038	ns	36	0.129	ns	85
	sST2	0.172	ns	29	**0.326**	**0.046**	**38**
	MPO	-0.198	ns	36	**0.340**	**0.002**	**84**
	IL-8	-0.113	ns	63	0.168	ns	73
Coagulation & Platelet activation	D-dimers	0.031	ns	51	0.221	ns	37
	sCD62p	0.110	ns	45	**0.445**	**0.001**	**53**
	sCD40L	-0.042	ns	50	0.172	ns	68
Parasite burden	PfHRP2	0.149	ns	63	-0.019	ns	87
	Pf cfDNA	-0.090	ns	36	**0.280**	**0.009**	**85**
	Parasitemia	ND	-	-	0.102	ns	86
Clinical parameter	PCV	**-0.256**	**0.040**	**62**	-0.017	ns	87
	Hemoglobin	**-0.313**	**0.010**	**64**	0.138	ns	83
	Platelets	0.103	ns	49	0.030	ns	85

UM: Uncomplicated Malaria; Ret+ CM: Retinopathy positive cerebral malaria;Rs: Spearman Rho; cfDNA: cell free DNA; sST2: soluble Suppression of Tumorigenicity 2; MPO: Myeloperoxidase; IL-8: Interleukin 8; PfHRP2: P. falciparum Histidine Rich Protein 2; PCV: Packed Cell Volume.Variables with a significant p-value < 0.05 are bolded and italiziced. P-values ≤ 0.05 are bolded; ND: Not determined; ns: not significant.

Supplemental Table S3.

Correlation of immune and clinical markers associated with anti-PS IgG.

Process	Variable	UM			Ret+ CM		
		Rho	p-value	N	Rho	p-value	N
NETosis, Inflammation & Cell Damage	Total cfDNA	**0.405**	**0.0004**	**73**	**-0.230**	**0.048**	**75**
	Host cfDNA	**0.407**	**0.017**	**63**	**-0.365**	**0.001**	**75**
	sST2	-0.115	ns	37	0.153	ns	45
	MPO	**0.497**	**0.005**	**70**	0.054	ns	75
	IL-8	-0.007	ns	70	-0.045	ns	67
Coagulation & Platelet activation	D-dimers	-0.035	0.039	31	0.228	ns	32
	sCD62p	**0.524**	**<0.0001**	**66**	**0.367**	**0.006**	**55**
	sCD40L	**0.336**	**0.005**	**67**	**0.253**	**0.034**	**70**
Parasite burden	PfHRP2	-0.189	ns	33	**-0.274**	**0.017**	**75**
	Pf cfDNA	0.215	0.068	34	0.069	ns	79
	Parasitemia	ND	-	-	0.104	ns	78
Clinical parameter	PCV	0.083	ns	71	-0.066	ns	79
	Hemoglobin	0.019	ns	71	0.113	ns	77
	Platelets	0.013	ns	36	0.100	ns	78

UM: Uncomplicated Malaria; Ret+ CM: Retinopathy positive cerebral malaria;Rs: Spearman Rho; cfDNA: cell free DNA; sST2: soluble Suppression of Tumorigenicity 2; MPO: Myeloperoxidase; IL-8: Interleukin 8; PfHRP2: *P. falciparum* Histidine Rich Protein 2; PCV: Packed Cell Volume.Variables with a significant p-value < 0.05 are bolded and italiziced. P-values ≤ 0.05 are bolded; ND: Not determined; ns: not significant.

Supplemental Table S4. Regression analysis of Anti-PS IgG with clinical outcomes/complications in Ret+ CM.

Variable	Odds Ratio	OR 95% CI	Beta	Beta 95% CI	Beta p-value	AUC	AUC p-value	N	N with complication
Severe Malaria Anemia	0.95	0.73 - 1.35	-0.06	-0.385 to 0.230	0.714	0.51	0.840	81	24
Respiratory Distress	1.26	0.97 - 1.84	0.23	-0.066 to 0.548	0.751	0.51	0.385	81	18
Jaundice	0.93	0.46 - 1.35	-0.08	-0.653 to 0.335	0.751	0.51	0.893	81	8
Death	0.93	0.63 - 1.40	-0.08	-0.542 to 0.281	0.705	0.50	0.991	81	12
